# Promotion, prevention and treatment interventions for mental health in low- and middle-income countries through a task-shifting approach

**DOI:** 10.1017/S204579602000061X

**Published:** 2020-08-03

**Authors:** Marianna Purgato, Eleonora Uphoff, Rakesh Singh, Ambika Thapa Pachya, Jibril Abdulmalik, Nadja van Ginneken

**Affiliations:** 1 WHO Collaborating Centre for Research and Training in Mental Health and Service Evaluation, Department of Neuroscience, Biomedicine and Movement Sciences, Section of Psychiatry, University of Verona, Verona, Italy; 2 Cochrane Global Mental Health, University of Verona, Verona, Italy; 3 Cochrane Common Mental Disorders, Centre for Reviews and Dissemination, University of York, York, UK; 4 Department of Community Health Sciences, Patan Academy of Health Sciences, Lalitpur, Nepal; 5 School of Public Health, Patan Academy of Health Sciences, Lalitpur, Nepal; 6 Department of Psychiatry, University of Ibadan, Ibadan, Nigeria; 7 Department of Primary Care and Mental Health, University of Liverpool, Liverpool, UK

**Keywords:** Low- and middle-income countries, prevention, primary mental health care, promotion, public mental health, task-shifting, treatment

## Abstract

Recently, mental health and ill health have been reframed to be seen as a continuum from health to ill health, through the stages of being asymptomatic ‘at risk’, to experiencing ‘mental distress’, ‘sub-syndromal symptoms’ and finally ‘mental disorders’. This new conceptualisation emphasised the importance of mental health promotion and prevention interventions, aimed at reducing the likelihood of future disorders with the general population or with people who are identified as being at risk of a disorder. This concept generated discussion on the distinction between prevention and treatment interventions, especially for those mental health conditions which lie between psychological distress and a formal psychiatric diagnosis. The present editorial aims to clarify the definition of promotion, prevention and treatment interventions delivered through a task-shifting approach according to a global mental health perspective.

## Introduction

The coronavirus pandemic has brought with it not only the physical sequelae of the viral infection but also rising levels of poverty, socioeconomic insecurity and physical and mental health problems worldwide. It is also postulated that the SARS-CoV2 virus may have neurological/neuropsychiatric impact on the brain (Holmes *et al*., 2020). Now more than ever, with rising mental health needs, it becomes even more important to find an effective solution to providing universal mental healthcare. Strategies also need to be rolled out to tackle the root social, economic, environmental and psychological causes of mental ill health to prevent mental disorders and promote wellbeing.

## The global burden of mental health

Mental, behavioural and neuropsychiatric disorders all feature in the top 30 causes of years lived with disability. The highest contributors are anxiety and depressive disorders, drug-use disorders and alcohol-use disorders (DALYs and Collaborators, 2018). Mental health and behavioural disorders contribute 7.4% of the global burden of disease in the world, more than, for example, tuberculosis (2.0%), HIV/AIDS (3.3%) or malaria (4.6%) (Whiteford *et al*., 2013). The contribution of major depressive disorders to worldwide disability-adjusted life years has increased by 37% from 1990 to 2010 and is predicted to rise further (Prince *et al*., 2007; Murray *et al*., 2012). Furthermore, self-inflicted injuries and alcohol-related disorders are likely to increase in the ranking of global disease burden due to the decline in communicable diseases and because of a predicted increase in war and violence. The disease burden due to Alzheimer's disease is also increasing, linked to the demographic transition towards an ageing population (Vos *et al*., 2012).

People living in low- and middle-income countries (LMICs) are exposed to a constellation of stressors that make them vulnerable to developing psychological symptoms and/or mental disorders, and a large gap between individuals in need of care and those who actually receive evidence-based interventions still exists (World Health Organization, 2010, 2015). Conceptualising mental health interventions is particularly relevant in settings with limited resources for interventions implementation.

## Conceptualising prevention and treatment in LMICs

Recently, mental health and ill health have been reframed to be seen as a continuum from health to ill health, through the stages of being asymptomatic ‘at risk’, to experiencing ‘mental distress’, ‘sub-syndromal symptoms’ (some symptoms suggested of a mental disorder but not sufficient to reach diagnostic categories) and finally ‘mental disorders’ (Patel *et al*., 2018). This new conceptualisation emphasised the importance of mental health promotion and prevention interventions, aimed at reducing the likelihood of future disorders with the general population or with people who are identified as being at risk of a disorder (Tol *et al*., 2015).

At the same time, this concept generated discussion on the distinction between prevention and treatment interventions for those mental health conditions which lie between psychological distress and a formal psychiatric diagnosis.

**Fig. 1.**
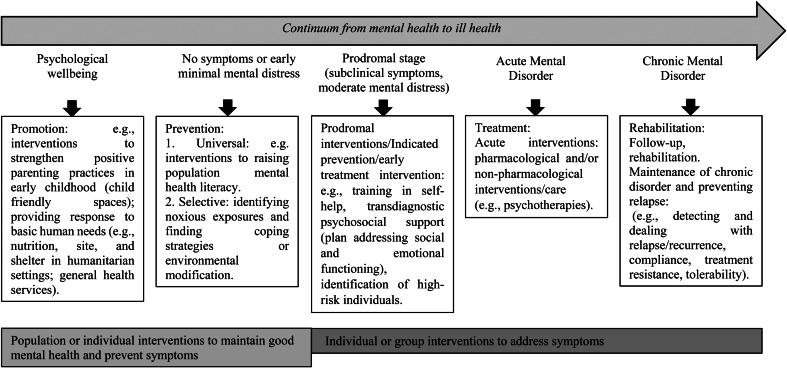
Examples of interventions according to the continuum from mental health to ill health (adapted from Patel *et al*., 2018).

The boundary between prevention and treatment is hard to draw in mental health. Figure 1 shows how staging has been conceptualised of mental health symptoms, together with where prevention and treatment interventions fit in. For example, wellbeing interventions are not just relevant to those who are asymptomatic as people with mental disorders can still work on and achieve a sense of wellbeing and quality of life and are therefore relevant across the stages (Patel *et al*., 2018).

Furthermore, these stages are not fixed or very well defined. Minimal or early distress is a state which can often fluctuate and may not be affecting someone's functioning much yet whereas people with prodromal symptoms may well start to affect their function. In practice, differentiating which populations in the study are in these categories is difficult as the populations are often mixed.

This issue is particularly important in LMIC settings, where it may not be affordable for mental health specialists (psychiatrists, psychologists) to administer diagnostic instruments (Saraceno, 2019; Barbui *et al*., 2020).

## The need for task-shifting

The gap between the individuals in need of mental health interventions and those who actually receive such care remains very large (World Health Organization, 2015). A study of 21 countries with the WHO Mental Health Surveys found that 52.6% of persons with depressive disorder in LMICs received any treatment in the past 12 months, and only 20.5% of persons with depressive disorder received minimally adequate treatment (Thornicroft *et al*., 2017). Furthermore, the quality of care received by many people, in particular those affected by severe mental disorders and disabilities, was poor in all countries and was often associated with abuses of their fundamental human rights (Patel *et al*., 2012). This is despite the existence of a range of cost-effective interventions in mental health care in LMICs (Tol *et al*., 2011; van Ginneken *et al*., 2013; Purgato *et al*., 2018*a*, 2018*b*; Barbui *et al*., 2020).

Major barriers to closing the treatment gap are the huge persistent scarcity of skilled human resources, large inequities and inefficiencies in resource distribution and utilisation, limited community awareness of mental health, poverty and social deprivation, and the significant stigma associated with psychiatric illness (Barber *et al*., 2019). Some papers have advocated for scaling up evidence-based services and for the task-shifting of mental health interventions to non-specialists as key strategies for closing the treatment gap (Patel *et al*., 2018). Moreover, the World Health Organization (WHO) developed the Mental Health Gap Action Programme Intervention Guide (mhGAP-IG) through a systematic review of evidence followed by an international participatory consultative process. The mhGAP-IG comprises straightforward, user-friendly, diagnosis-specific clinical guidelines for providing evidence-based practices for non-specialised health care providers. The mhGAP may be adapted for national and local needs, and consider the task-shifting approaches a promising strategy for improving mental health care delivery (World Health Organization, 2015). Task-shifting entails the shifting of tasks, typically from more to less highly trained individuals to make efficient use of these resources, allowing all providers to work at the top of their scope of practice. This includes primary care health workers (PHWs) and community workers (CWs). PHWs are first-level health providers who have received general health training rather than specialist mental health training and can be based in a primary care clinic or in the community. Cadres included are professionals (doctors, nurses and other general paraprofessionals) and non-professionals (such as trained lay health providers). PHWs do not include, for example, psychiatrists, psychologists, psychiatric nurses or mental health social workers. CWs such as teachers and community-level workers who have no background health training, but who may perform a particular mental health function within their role, are a further human resource employed in delivering promotion, prevention and treatment interventions (Patel *et al*., 2007).

**Fig. 2.**
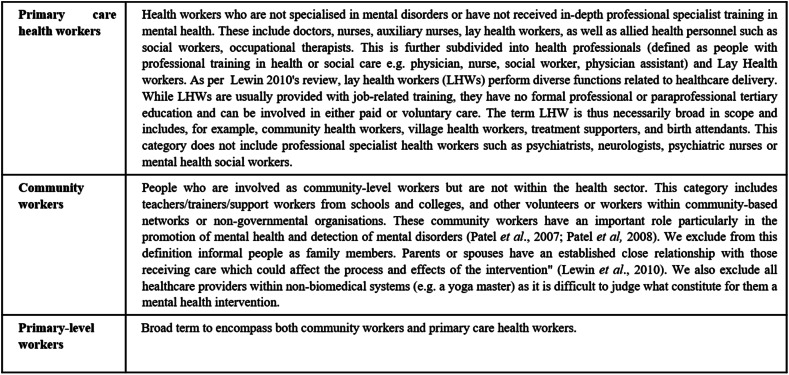
Definitions of workers involved in the task-shifting approach.

The differences in the organisation of mental health services between LMICs and high-income countries (HICs), with poorer countries having little or no mental health service structures in primary care or the community, means that the problem of providing mental health care is different in such settings. PWs may need to work with little or no support from specialist mental health services and fewer options for referral. Consequently, PWs interventions might be expected to function differently in many LMICs compared with HICs. In LMICs, PHWs and CWs have been employed in various services, including those delivered by governmental, private and non-governmental organisations in clinics, half-way homes, schools and communities. For example, lay health workers have been involved in supporting carers, befriending, ensuring adherence and delivering simple mental health interventions (Tol *et al*., 2020). Nurses, social workers and CWs may also take on follow-up or educational/promotional roles (Araya *et al*., 2003; Chatterjee *et al*., 2003; Chatterjee *et al*., 2008). In addition, doctors with general mental health training have been involved in the identification, diagnosis, treatment and referral of complex cases (Patel *et al*., 2008). Teachers and other educational support staff have been an important resource for child mental health care (Dybdahl, 2001; Gordon *et al*., 2008; Shen *et al*., 2018) and for the delivery of prevention interventions (Ager *et al*., 2011). The task-shifting approach is being used across a wide range of mental conditions in LMICs and has increasing evidence of being effective (van Ginneken *et al*., 2013 – update in progress), though still only a small percentage of psychological interventions in LMICs actually include non-specialists as providers (Barbui *et al*., 2020) (Fig. 2).

## Definition of mental health promotion and prevention interventions

Promotion is an approach aimed at strengthening positive aspects of mental health and psychosocial wellbeing, and is focused on empowering people to live healthy lives (e.g. by facilitating healthy lifestyles through policies, such as providing nutritious foods in school canteens or opportunities for physical exercise in accessible locations), rather than health being the sole domain of health professionals (National Research Council and Institute of Medicine, 2009). It includes – for example – components to foster pro-social behaviour, self-esteem, coping, decision-making capacity, but also universal interventions such as social and economic interventions to improve people's social determinants of health which would impact on their wellbeing. Prevention is an approach aimed at reducing the likelihood of future disorder in the general population or for people who are identified as being at risk of a disorder (Eaton, 2012; Tol *et al*., 2015). Prevention is further subdivided on the basis of the population targeted, into universal, selective and indicated (National Research Council and Institute of Medicine, 2009). Universal prevention, which includes strategies that can be offered to the whole population including individuals who are not at risk, based on the evidence that it is likely to provide some benefit to all (reduce the probability of disorder), clearly outweighs the costs and risks of negative consequences. Examples of common universal prevention interventions include the community-wide provision of information on positive coping methods (IASC, 2007) to help people feeling safe and hopeful, protection against human rights violations (e.g. gender-based violence), community-wide efforts to improve livelihoods as a key protective factor for mental health.

Selective prevention refers to strategies that are targeted to subpopulations identified as being at elevated biological, social or psychological risk for a disorder but who are asymptomatic or have very minimal symptoms. These interventions involve human, supportive and practical help covering both a social and a psychological dimension. They work through communication (asking about people needs and concerns; listening to people and helping them to feel calm), practical support (i.e. providing meals or water) and with a psychological approach including teaching stress management skills and helping people to cope with problems (World Health Organization, 2011); facilitation of community support for vulnerable individuals by activating social networks and communication; structured cultural and recreational activities supporting the development of resilience (National Research Council and Institute of Medicine, 2009), such as traditional dancing, art work, sports and puppetry. These activities may take place in equipped settings with the aim of increasing the children's sense of connectivity and safety (Tol *et al*., 2011).

Indicated prevention includes strategies that are targeted to individuals who are identified (or individually screened) as having detectable signs or symptoms which can foreshadow, precede and may sometimes – if left unaddressed – lead to a full diagnosable mental disorder based on an individual assessment. These interventions to prevent mental disorders may be delivered at individual or group level, in a variety of settings (antenatal and postnatal visits, home visits, community settings, schools, etc.). These interventions include psychosocial support for persons with subclinical levels of mental disorders (Purgato *et al*., 2019*a*), such as mentoring programmes aimed at children with behavioural problems; psychological first aid for people with heightened levels of psychological distress after exposure to severe stressors, loss or bereavement (Tol *et al*., 2015). This includes facilitator-guided self-help group interventions, as for example the WHO Self-Help Plus (Epping-Jordan *et al*., 2016; Purgato *et al*., 2019*a*).

Unlike HICs, in LMICs, factors as the socioecology of poverty, malnutrition, political conflicts, lack or poor implementation of mental health policy, poor governance in mental health and health systems, and lower priority for mental health influence the epidemiology, outcomes and treatment strategies of mental health problems (Yasamy *et al*., 2011; Baingana *et al*., 2015).

## Definition of treatment interventions

Treatment interventions are delivered to people who have a diagnosed mental disorder. However, sometimes, these treatment interventions, particularly psychological or psychosocial interventions, are also considered as effective treatments for those population groups that may receive ‘indicated prevention’ interventions in the category above. From the 2018 Lancet Commission on global mental health (which reconceptualised mental illness symptoms along a transdiagnostic staged spectrum), there is some evidence that treatments for mental disorders can overlap and be as effective for those with prodromal symptoms as for those with a diagnosable mental disorder (Patel *et al*., 2018).

Treatment interventions include various forms of psychotherapy and/or pharmacological treatment. In addition, treatment interventions may include broader interventions sometimes delivered by PHWs or CWs (and sometimes by specialist psychiatric nurses) such as training in self-help interventions, informal support, transdiagnostic psychosocial support (individualised plan addressing social and emotional functioning and problems) and high-risk individual identification which may be particularly relevant to those who have detectable subthreshold signs and symptoms of mental illness (van Ginneken *et al*., 2013).

Long-term interventions are important to help rehabilitate people after acute mental disorders, maintain stable mental health for those with chronic mental disorders and prevent recurrence or relapse. These could include roles in follow-up or rehabilitation of people with chronic severe mental disorders, and roles in detecting and dealing with relapse/recurrence, compliance issues, treatment resistance, side effects of treatment or psychosocial problems (Patel *et al*., 2018).

These may be individual or combined interventions, delivered either as a simple contained group of sessions, or as a complex collaborative care provision following a stepped care protocol or a shared care between primary care and specialist care (van Ginneken *et al*., 2013; Barbui *et al*., 2020).

## Challenges of delivering mental health interventions in LMICs

Despite the conceptual similarities and growing evidence for mental health promotion, prevention and treatment interventions may share conceptual similarities across the world and have growing evidence, delivering these interventions in LMICs is bound with several challenges. The acceptability of interventions might also be different, especially as for distressed participants who do not present an established psychiatric diagnosis dealing with their psychological distress may not be a high priority as dealing with other social or health issues. Participants (and their families) with a mental disorder, by contrast, may recognise that dealing with psychological problems is a high priority and a pre-requisite for optimal social functioning, thus showing more compliance and participation in psychological interventions.

Many LMICs either lack or are poor in implementation of mental health policies, programmes and interventions and have difficult access to mental health care (Alloh *et al*., 2018). A key factor attributing to mental health issues in LMICs is the discrimination against people suffering from mental illnesses where often they are labelled, exempted and even abused (Alloh *et al*., 2018). Henceforth, people in LMICs are often reluctant to seek mental healthcare services to avoid the circumstances where they are socially discriminated. The condition is further aggravated in many LMICs where people identified with mental health problems experience stigma even during treatment, which in turn leads to poor care, delay in seeking health services or non-adherence to treatments (Alloh *et al*., 2018). ‘For an instance, it is a very common myth that people suffering from mental illness rarely get recovered in South Western Nigeria’ (Orngu, 2015).

Additionally, the coordination and management of mental health interventions in humanitarian settings including conflicts, disasters, epidemic and pandemic may present major challenges. For example, despite an increase in the incidence of mental health problems during armed conflicts, earthquakes, epidemics and famine in countries like Nepal, Haiti and Ethiopia, the limited resources are diverted to areas other than mental health (Rathod *et al*., 2017).

There may also be many different socio-economic factors which influence the burden of mental health. In many LMICs, social factors such as poverty, gender, urbanisation, internal migration and lifestyle changes are moderators of the magnitude of mental health problems (Rathod *et al*., 2017; Wainberg *et al*., 2017). Furthermore, low levels of knowledge regarding mental health problems have been suggested as an important factor that delays the interventions' onset (Henderson *et al*., 2013).

Finally, the resources for delivery and training, and the types of cadres of health workers involved increase heterogeneity across interventions, which become difficult to compare. Training, supervision and competency assessment of those delivering these interventions have also traditionally not been priorities in LMIC due to scarce human and financial resources (though these have become increasingly addressed features of LMIC trials) (Kakuma *et al*., 2011) and limited dissemination and implementation research capacity (Wainberg *et al*., 2017).

## Challenges in conducting public mental health research in LMICs

Despite research in global mental health rapidly growing, with rigorous studies implemented in LMIC settings, there remain several research challenges to be addressed. Mental ill health is globally recognised as one of the major public health problems yet mental health care and promotion/prevention are less prioritised in many LMICs (Alloh *et al*., 2018). Furthermore, there are various difficulties that are faced by mental health researchers in LMICs including lack of good mental health research governance, lack of funding, shortage of trained personnel to carry out mental health research, unequal distribution of mental health research capacity, difficulty in training due to weaker institutional infrastructure, constraints on investigators' time owing to healthcare delivery and teaching responsibilities, absence of a strong research ‘culture’, poor peer networks and collaborations (The Academy of Medical Sciences, 2008; Yasamy *et al*., 2011). Moreover, there are other practical problems and context-dependent issues that hinder mental health research in LMICs. For example, low mental health literacy among the larger research community and frequent migration make large-scale intervention trials and prospective studies a challenge (Yasamy *et al*., 2011).

Given the magnitude of the burden of mental disorders, although treatment intervention alone will not be enough to close the mental health gap in LMICs, mental health promotion and prevention of mental illness are at an incipient stage in most LMICs (Wainberg *et al*., 2017). Although difficult to achieve in LMICs, decreasing structural inequality, stigma and social discrimination is an important prevention intervention targeted towards mental illnesses. Current evidence is insufficient to determine what prevention interventions are effective and feasible for decreasing stigma in LMICs, how best to target key groups such as health care staff, and how to adapt such interventions in specific contexts (Wainberg *et al*., 2017). One of the complexities with research interventions delivered in LMICs is that asymptomatic, prodromal and/or disordered populations overlap within the same experimental study. There is variation in the categorising of interventions and/or population groups as belonging to the treatment or various prevention categories. In practical terms, it means that experimental studies may include participants showing no distress, some psychological distress and/or participants with a formal psychiatric diagnosis. This is due often to not having the setting, tools, manpower or not felt appropriate to select people based on screening tools, but rather based on situational settings – a much more immediate and tangible inclusion criterion particularly in difficult settings like war-torn or highly deprived settings. Mixed population groups are thus likely to increase heterogeneity, as the clinical response and compliance to interventions may vary. In this scenario, subgroup analyses based on participant symptom stage may be a strategy to evaluate interventions' efficacy.

The ‘grey area’ between treatment and prevention, i.e. the indicated prevention, is often difficult to categorise as their aims can be to either treat participants to reduce their symptoms or help them recover, or to prevent the development of mental disorder. Whilst categorising these interventions to decide which of the parallel systematic reviews on treatment and prevention interventions (both ongoing) they would fit in, we divided these studies according to these expected aims and outcomes. Studies where the intervention aim was to achieve recovery or symptom improvement were included in the treatment review (van Ginneken *et al*., 2013 – update in progress). Those aimed at preventing mental disorders went into the prevention review (Purgato *et al*., 2020). Several studies were difficult to discern and needed to be included in both reviews due to uncertainty of mixed populations. Once these reviews are completed we may be able to produce more specific guidance on whether this strategy worked and how.

Furthermore, the choice of control group is relevant for research in LMICs and may have clinical implications. In many LMICs, participants suffer from long-lasting and even chronic conditions because they lack the possibility of receiving appropriate evidence-based treatments (Purgato *et al*., 2019*b*). Despite the waiting list as a control condition has been criticised because of limiting participants seeking care for their mental condition elsewhere because they are waiting for the intervention (Cuijpers and Cristea, 2016; Cuijpers *et al*., 2018), this is less of a concern in many LMICs, in which often the alternative is simply not receiving care at all. Even the control group defined as treatment as usual (TAU) may vary according to populations and contexts, to the point that being in the TAU condition sometimes corresponds to not getting treatments at all and differentiating TAU from no treatment or from waiting list control might become difficult.

## Conclusions

We do not intend to provide a conclusive or simplistic framework for categorizing mental health interventions in LMICs. However, clarifying key concepts of relevance to public mental health and how it is intertwined with task-shifting to expand universal access, may help both researchers and practitioners in the design, assessment and implementation of evidence-based interventions.
